# Instrumental Activities of Daily Living in Neurocognitive Disorders: Determinants and Clinical Implications for Health Promotion

**DOI:** 10.3390/brainsci15040417

**Published:** 2025-04-19

**Authors:** Anna Tsiakiri, Spyridon Plakias, Christos Kokkotis, Pinelopi Vlotinou, Sotiria Kyriazidou, Georgios Giarmatzis, Stylianos Kallivoulos, Aikaterini Terzoudi, Dimitrios Tsiptsios, Souzana Merai, Chrysoula Emmanouilidou, Christos Kariotis, Anna Kanidou, Nikolaos Aggelousis, Konstantinos Vadikolias, Foteini Christidi

**Affiliations:** 1 Dementia Day Center, Department of Neurology, School of Medicine, Democritus University of Thrace, 68100 Alexandroupolis, Greece; atsiakir@med.duth.gr (A.T.); sokyriaz@med.duth.gr (S.K.); kallivoulosmd@gmail.com (S.K.); terzoudi@med.duth.gr (A.T.); souzanamerai@gmail.com (S.M.); xemmanouilidou38@gmail.com (C.E.); ckariotes@gmail.com (C.K.); anna_kanidou@hotmail.com (A.K.); kvadikol@med.duth.gr (K.V.); 2 Department of Physical Education and Sport Science, University of Thessaly, 42100 Trikala, Greece; spyros_plakias@yahoo.gr; 3 Department of Physical Education and Sport Science, Democritus University of Thrace, 69100 Komotini, Greece; ckokkoti@affil.duth.gr (C.K.); ggiarmat@phyed.duth.gr (G.G.); nagelous@phyed.duth.gr (N.A.); 4 Department of Occupational Therapy, University of West Attica, 12243 Athens, Greece; pvlotinou@uniwa.gr; 5 3rd Department of Neurology, Aristotle University of Thessaloniki, 54124 Thessaloniki, Greece; tsiptsios.dimitrios@yahoo.gr

**Keywords:** instrumental activities of daily living, neurocognitive disorders, cognition, problem solving, balance, caregivers’ burden, health promotion

## Abstract

**Background/Objectives**: Instrumental Activities of Daily Living (IADL) are the key indicators of the autonomy and functional ability in older adults with neurocognitive disorders (NCDs). However, the specific predictors of IADL performance across the NCD spectrum remains insufficiently characterized. This study aimed to identify the cognitive, motor, and caregiver-related determinants of the IADL in individuals with minor and major NCDs. **Methods**: A cross-sectional study was conducted involving 117 participants referred from a university-based dementia clinic. Standardized tools were administered to evaluate their IADL performance (Lawton IADL Scale), cognition [Addenbrooke’s Cognitive Examination III (ACE-III)], Functional Cognitive Assessment Scale (FUCAS)], motor functions, balance, and mobility [Tinetti Test, Timed Up and Go (TUG)], emotional status [Geriatric Depression Scale (GDS)], neuropsychiatric symptoms [Neuropsychiatric Inventory (NPI)], and caregiver burden [Zarit Burden Interview (ZBI)]. Multiple regression analyses were performed to identify the significant predictors of IADL performance. **Results**: In the total sample (n = 117), the IADL performance was significantly predicted via ACE-III, FUCAS, and Tinetti-balance (adjusted R^2^ = 0.729). In the minor NCD group (n = 41), the significant predictors included sex, FUCAS, GDS, Tinetti-balance, and TUG (adjusted R^2^ = 0.725). In the major NCD group (n = 76), ACE-III, FUCAS, and Tinetti-balance remained the significant predictors (adjusted R^2^ = 0.634). Female sex and a worse profile on the other variables were associated with lower IADL scores. **Conclusions**: Global cognitive decline, executive dysfunction in everyday problem-solving situations, and balance impairment are the key determinants of IADL performance across both minor and major NCDs. Female sex and depressive symptoms further predicted the IADL performance in the minor NCD group. These findings highlight the need for multidisciplinary assessment and intervention strategies to promote health and autonomy and preserve the functional independence in older adults with NCDs.

## 1. Introduction

Neurocognitive disorders (NCDs) encompass a spectrum of cognitive impairments according to the fifth edition of the Diagnostic and Statistical Manual of Mental Disorders (DSM-5), and aging remains the most prominent risk factor across all the etiological subtypes. The term “NCDs” was introduced to replace the previous classification of “Dementia, Delirium, Amnestic, and Other Cognitive Disorders” [1,2]. This change reflects a conceptual shift from viewing these conditions as categorical, end-stage syndromes toward a continuum of cognitive decline, encompassing both mild and major levels of impairment. Ιn DSM-5, Mild Cognitive Impairment (MCI) aligns with the diagnostic category of “minor” NCDs, representing a clinically significant decline in cognitive function that does not yet impair independence in everyday functioning. While MCI is often associated with neurodegenerative processes, it is not pathognomonic of Alzheimer’s disease and can result from various etiologies. The new term emphasizes the neurobiological underpinnings of cognitive decline and aims to facilitate early detection and intervention, especially in the preclinical stages of diseases such as Alzheimer’s disease (AD) [3,4]. Unlike DSM-4, which primarily relied on the presence of memory deficits, DSM-5 broadens the scope to include impairments in six cognitive domains, including executive function and social cognition, and allows for diagnosis even in the absence of memory impairment, aligning better with clinical and research advances [5]. Furthermore, the term “NCDs” provides a diagnostically inclusive framework for a wide range of etiologies (e.g., vascular, traumatic, substance related), and supports clinical decision making in line with the dimensional models of psychiatric classification [6]. Although the term “NCDs” may be less widely recognized in some contexts, it reflects the DSM-5 shift away from “dementia” to a spectrum-based, etiologically neutral classification that better accommodates both clinical diversity and early-stage cognitive decline [2,3,6].

Greece, in particular, is among the most rapidly aging countries in Europe [7], with estimations to become the most elderly nation on the continent by 2050 according to the Organisation for Economic Co-operation and Development. The current estimates suggest that approximately 200,000 individuals are living with NCDs in Greece [7]. Considering that each patient significantly impacts the lives of two to three family caregivers, it is evident that nearly 1.5 million Greek citizens are directly affected by this condition. The annual economic burden of NCDs in Greece is estimated to be around EUR 3 billion [8].

A core feature of NCDs, even in their earliest stages, is a decline in functional independence, most notably in the Instrumental Activities of Daily Living (IADL), such as managing finances, medication, shopping, or using transportation. These activities require the integration of multiple cognitive and motor domains and are typically the first to deteriorate in the presence of cognitive impairment [2]. As global populations continue to age, the incidence of NCDs is expected to rise significantly, with advancing age being the most prominent risk factor for both cognitive decline and functional disability [9]. Therefore, IADL performance emerges as a sensitive clinical marker of early autonomy loss and is increasingly recognized as a key outcome in the research and care planning for aging populations with cognitive disorders. Moreover, maintaining functionality is a central component of “healthy aging”, while “usual aging” typically involves a decline in functional capacity and, consequently, in the quality of life [10,11,12]. According to the World Health Organization’s International Classification of Functioning, Disability and Health model, disability and functionality result from interactions between personal and environmental factors, and these can be evaluated through specific activities [13]. In addition, the assessment of disability must be contextualized within each sociocultural setting [9,14].

The importance of the sociocultural context in understanding functional disability has been previously highlighted [15], with significant regional variations in disability levels and the lowest levels in Northern and Western Europe. Disability, by definition, often entails a loss of autonomy and increased dependence on others for care [16]. This pattern is commonly observed in the progression of NCDs, from minor to major NCDs [17]. Furthermore, such dependence occurs not only in AD but also in other neuropathologies, including frontotemporal dementia, vascular dementia, Parkinson’s disease dementia, and Lewy body dementia [18]. Emerging evidence highlights that IADL performance is influenced by a combination of cognitive, motor, and psychosocial factors [19,20,21]. Moreover, caregivers’ burden, especially in the early stages of NCDs, may indirectly affect patients’ autonomy and has been identified as a key psychosocial factor in the progression of functional decline [12,15,19].

Taking all the above into consideration, it becomes evident that the functional ability in the IADL is a multidimensional construct influenced by cognitive, motor, and psychosocial factors. However, limited research has explored the specific determinants of the functional decline in individuals with NCD spectrum, especially in Greece. Therefore, we investigated the key cognitive, motor, emotional, neuropsychiatric, and caregiver-related variables that affect IADL performance, with the aim of identifying the indicators of functional decline and informing targeted interventions to promote health, preserve autonomy, and enhance the quality of life of older adults with NCDs.

## 2. Materials and Methods

We conducted a cross-sectional study to evaluate the determinants of the IADL in individuals with NCDs. All the data were collected in accordance with the Declaration of Helsinki and in compliance with the Ethical Committee of the University Hospital of Alexandroupolis (ΔΣ1/Θ68/06-04-2020). Written informed consent was obtained from all the participants. In cases involving individuals with dementia, consent was additionally obtained from their caregiver and/or legal representative. The consent form clearly outlined the purpose of the study, its expected duration, and the potential benefits for cognitive and psychological functioning. The participants were informed that their participation was voluntary, and that they could withdraw at any time without affecting their medical care or legal rights. The confidentiality procedures were explained, including how their health data would be handled and the possibility that the Institutional Review Board or other regulatory authorities might review their records to ensure the study’s integrity. All the questions raised by the participants or their caregivers were addressed prior to participation. The data were analyzed anonymously.

### 2.1. Participants

The data from 117 individuals (aged 60–89 years) with minor or major NCDs were included in the study. The participants were recruited from the Outpatient Dementia Center of the University General Hospital of Alexandroupolis, which primarily serves the region of Eastern Macedonia and Thrace, Greece. The sample comprised both urban and rural residents. The demographic information collected included age, sex, education level, and disease duration. Detailed medical histories were also obtained, including cardiovascular, metabolic, and neurological conditions, as well as the presence of affective disorders.

#### Inclusion/Exclusion Criteria

A semi-structured interview was conducted to collect demographic and clinical data, including medical diagnoses, histories of cardiovascular, metabolic, and neurological conditions, and the presence of affective disorders. Individuals with NCDs underwent a comprehensive neurological examination, neuropsychological assessment, and gait evaluation. Their caregivers contributed additional information through cognitive and functional assessments of the patients, as well as by completing a burden inventory measuring their own caregiving strain.

Forty-one individuals met the diagnostic criteria for minor NCDs as defined by the DSM-5 [22]. These criteria include the following: (a) a decline in cognitive functions, either self-reported or observed by a family member or clinician; (b) cognitive impairment relative to the individual’s age, confirmed through formal neuropsychological assessment; (c) objective evidence of gradual cognitive decline beyond what is expected with normal aging, without meeting the criteria for dementia; (d) preserved overall cognitive abilities and daily functioning; and (e) the absence of a prior dementia diagnosis or other conditions (e.g., depression, delirium, intoxication, or psychosis) that could account for the observed impairment.

Seventy-six individuals met the diagnostic criteria for major NCDs as defined by DSM-5 [22]. The criteria include the following: (a) a significant cognitive decline from a previous level of performance, as reported by the individual, a family member, or a clinician; (b) objective evidence of substantial cognitive impairment in one or more domains (e.g., memory, executive function, attention, language, visuospatial skills) confirmed through a standardized neuropsychological assessment; (c) cognitive deficits that interfere with the individual’s ability to perform the instrumental or basic activities of daily living independently; (d) progressive deterioration in cognitive function beyond what is expected with normal aging; and (e) exclusion of alternative explanations for the impairment, such as delirium, major psychiatric disorders (e.g., depression or psychosis), substance intoxication, or other medical conditions.

The exclusion criteria included cognitive deficits resulting from secondary factors, as confirmed through laboratory tests such as vitamin B12 and folate levels, as well as thyroid function tests. Participants with structural brain abnormalities detected via conventional MRI—such as territorial infarction, intracranial hemorrhage, brain tumors, hydrocephalus, or traumatic brain injury—were also excluded. Additionally, individuals who were unable to walk independently and required assistive devices for mobility were not included in the study, as motor and balance assessments (i.e., the Tinetti Test and the Timed Up and Go Test) were the key variables in the analysis, and non-ambulatory participants would not be able to complete these assessments reliably.

### 2.2. Measures

In this study, a series of standardized assessment tools were used to evaluate multidimensional factors—including cognitive, motor, functional, and emotional domains—in individuals with NCDs. These instruments were selected to assess IADL, overall cognitive performance, executive function in everyday problem-solving situations, balance, gait, mobility, patients’ emotional status and neuropsychiatric symptoms, and caregiver burden. By incorporating multiple validated measures, the study aimed to offer a comprehensive understanding of the interplay between cognition, motor function, and daily living abilities in individuals with NCDs. The following section describes each of the assessments utilized in this study.

IADL: The Lawton IADL Scale [23,24] is a widely used measure for assessing an individual’s ability to perform complex daily tasks essential for independent living. It evaluates eight functional domains: telephone use, shopping, food preparation, housekeeping, laundry, transportation, medication management, and financial management. Scores range from 0 (completely dependent) to 8 (fully independent). Although primarily based on self-reporting, the IADL scale offers valuable insights into an individual’s functional capacity and can inform interventions aimed at preserving or enhancing independence.

Cognition: The Addenbrooke’s Cognitive Examination III (ACE-III) [25,26] is a comprehensive cognitive screening instrument designed to assess multiple cognitive functions, including memory, language, attention, and visuospatial abilities. It is widely utilized by healthcare professionals—particularly neurologists and geriatricians—for the detection and monitoring of the cognitive impairments associated with conditions such as AD. The ACE-III evaluates five core cognitive domains: attention and orientation, memory, verbal fluency, language, and visuospatial abilities. It consists of a series of structured tasks and questions, with a maximum possible score of 100. Higher scores reflect better cognitive functioning, and lower scores indicate cognitive impairment.

The Functional Cognitive Assessment Scale (FUCAS) [27] is a cognitive–behavioral tool developed to evaluate the executive function in everyday problem-solving situations among individuals with dementia and those in preclinical stages. Unlike many existing assessments that rely on self-reports or caregiver input, FUCAS directly measures the executive function through the administration of structured tasks. This scale assesses seven key executive function parameters across six daily activities, including telephone communication, shopping, orientation in place, medication management, personal hygiene, and dressing. The executive function parameters measured include problem awareness, working memory, planning, time distribution, step sequencing, accuracy, and goal maintenance. FUCAS provides subscores for each executive function parameter and a total score ranging from 42 to 126, with higher scores indicating greater cognitive impairment.

Motor functions, balance, and mobility: The Tinetti Test, also known as the Performance-Oriented Mobility Assessment (POMA) [28], is a widely used clinical instrument for evaluating the balance and gait in older adults and for predicting their fall risk. It consists of two subscales: a balance subscale, which assesses static and dynamic balance through tasks such as sitting, rising from a chair, standing, turning, and responding to external perturbations; and a gait subscale, which evaluates the step length, step symmetry, step continuity, trunk stability, and gait initiation. Each subscale is scored independently, with the balance component typically rated on a 16-point scale and the gait component on a 12-point scale, yielding a maximum total score of 28. Higher scores reflect better balance and mobility, whereas lower scores are indicative of a greater fall risk. Although specific cut-off values vary across studies, scores below 19–21 are generally associated with an increased likelihood of falling.

The Timed Up and Go (TUG) Test [29] is a widely employed clinical assessment used to evaluate the mobility, balance, and fall risk in older adults. It measures the time required for an individual to stand up from a chair, walk a distance of three meters (10 feet), turn around, walk back, and sit down again. The test is performed at a normal walking pace. A completion time of ≤10 s is typically considered within normal limits, while times ≥12–14 s are generally indicative of an increased risk of falls. In addition to the total time, observations of the gait stability, stride length, and balance control during the test can yield further insights into neuromuscular impairments.

Patients’ emotional status and neuropsychiatric symptoms: Depressive symptoms were evaluated using the Geriatric Depression Scale (GDS) [30,31]. Neuropsychiatric changes were assessed through the Neuropsychiatric Inventory (NPI) which is administered to caregivers [32].

Caregivers’ burden: The Zarit Burden Interview (ZBI) [33] is one of the most widely utilized instruments for assessing the caregiver burden, particularly among those caring for individuals with dementia. It measures the emotional, financial, and physical strain experienced by caregivers through 22 self-reported items, each rated on a 5-point Likert scale. The total score ranges from 0 to 88, with higher scores indicating greater caregiver burden. A score of 61 or above typically reflects a high level of caregiver burden, while lower scores suggest a mild to moderate burden.

### 2.3. Statistical Analysis

The continuous variables are presented as mean ± standard deviation (SD) while the categorical variables are reported as absolute frequencies. The assumption of normality was assessed prior to further analysis. Multiple regression analysis was performed to examine the contribution of patients’ demographics, disease duration, cognitive and neuropsychiatric profile, motor functions, and caregivers’ burden to the total IADL score. The IADL total score was used as the dependent variable, and the following variables were included as independent predictors: age, sex, education, disease duration, ACE-III total score, FUCAS, GDS, NPI total score, TUGG, TINETTI-gait, and TINETTI-balance, and caregivers’ ZBI. The analysis was conducted for the entire sample, and subsequently for the subgroups of individuals with minor and major NCDs (MinorNCD and MajorNCD, respectively). The multicollinearity was evaluated using the correlation matrix and diagnostic indices (i.e., tolerance, variance inflation factor). The statistical significance was set at *p* < 0.05. All the analyses were conducted using the IBM SPSS, version 29.

## 3. Results

### 3.1. Group Characteristics

We included 117 patients with MinorNCD (n = 41) and MajorNCD (n = 76). The demographic, clinical, cognitive, neuropsychiatric, and motor characteristics of the participants are presented in Table 1. Significant differences were observed between the two groups in terms of age, educational level, ACE-III total score, FUCAS score, NPI total score, TUG score, IADL total score, and caregivers’ ZBI score. Patients with MajorNCD were older and had lower educational attainment compared to those with MinorNCD, and they also demonstrated a poorer performance across the cognitive, neuropsychiatric, and motor measures.

### 3.2. Determinants of IADL

Patients’ ACE-III total score (B = 0.041; *p* < 0.001), FUCAS score (B = −0.050; *p* < 0.001), and TINETTI-balance score (B = 0.208; *p* < 0.001) were significant predictors of the IADL total score (F_3,116_ = 105.103; *p* < 0.001), accounting for 72.9% of the variance (Table 2). The regression B coefficients represent the expected change in the IADL total score associated with a one-unit increase in each predictor. A higher general cognitive status (indicated by a higher ACE-III total score), better executive functions in everyday problem-solving situations (reflected by a lower FUCAS score), and a greater balance ability (indicated by a higher TINETTI-balance score) were all associated with increased independence in the IADL (reflected by a higher IADL score).

When the patients with minor NCDs were analyzed separately, sex (B = 1.119; *p* = 0.007), the FUCAS score (B = −0.087; *p* < 0.001), GDS score (B = −0.207; *p* = 0.007), TINETTI-balance score (B = −0.329; *p* < 0.001), and TUG score (B = −0.185; *p* = 0.007) significantly predicted the IADL total score (F_3,40_ = 22.119; *p* < 0.001), explaining 72.5% of the variance. Greater independence in the IADL (indicated by a higher IADL score) was associated with the female sex, better executive functions in everyday problem-solving situations (reflected by a lower FUCAS score), fewer depressive symptoms (indicated by a lower GDS score), superior balance (reflected by a higher TINETTI-balance score), and faster completion times in the TUG test (reflected by a lower TUG score).

When the patients with major NCDs were analyzed separately, the ACE-III total score (B = 0.028; *p* = 0.027), FUCAS score (B = −0.052; *p* < 0.001), and TINETTI-balance score (B = 0.124; *p* = 0.045) significantly predicted the IADL total score (F_4,75_ = 44.335; *p* < 0.001), accounting for 63.4% of the variance. Greater independence in the IADL (indicated by a higher IADL score) was associated with better general cognitive functioning (reflected by a higher ACE-III total score), superior executive functions in everyday problem-solving situations (indicated by a lower FUCAS score), and better balance (reflected by a higher TINETTI-balance score).

## 4. Discussion

This study aimed to identify the key determinants of IADL performance across the NCD spectrum. In summary, up to 72.9% of the variance in the IADL performance was explained by reference to the cognitive, executive, and motor variables, including the ACE-III, FUCAS, and TINETTI-balance scores. Subgroup analyses of individuals with minor and major NCDs further revealed distinct sets of significant predictors within each group.

### 4.1. IADL and Cognition in NCDs

The findings of this study suggest that the general cognitive status plays a crucial role in IADL performance, particularly among individuals with major NCDs. The IADL require more complex neuropsychological processing than the basic activities of daily living (BADL), rendering them more vulnerable to decline associated with cognitive deterioration [34]. Individuals with mild AD exhibit substantial impairments in IADL, and 45–65% of them are unable to perform routine tasks at the baseline whereas up to 85% require assistance after three years [34]. However, not all studies report strong associations between cognitive measures and IADL performance. For instance, Martyr et al. [35] found that attention, rather than executive functions or memory, was a significant predictor of the BADL in individuals with dementia. These findings indicate that other cognitive domains may play a crucial role in functional abilities [36]. Moreover, the differences in study methodologies, sample characteristics, and assessment tools may account for the variability observed across the studies.

### 4.2. IADL and Executive Function in Everyday Problem-Solving Situations in NCDs

The study found that higher executive functions in everyday problem-solving situations are associated with greater independence in IADL, in both the minor and major NCD groups. The relationship between cognitive functions and functional abilities has been extensively investigated, with growing evidence indicating that executive functioning and problem solving play a critical role in the performance of IADL. These tasks, such as managing finances, grocery shopping, and adhering to medication, require complex cognitive processes such as planning, decision making, and problem solving. Research has consistently demonstrated that impairments in executive functions significantly affect an individual’s ability to carry out these activities, thereby reducing their functional independence [37,38,39,40,41]. Executive dysfunction, in particular, has been closely associated with limitations in IADL. Previous studies have underscored the critical role of executive abilities in daily task management, showing that greater executive impairment correlates with poorer IADL performance [42]. Similarly, impairments in both executive functions and memory have been strongly linked to difficulties in executing the IADL [41]. Furthermore, individuals exhibiting more pronounced executive dysfunction tend to experience greater limitations in both the BADL and IADL [43], with the latter being more substantially affected. Moreover, individuals with MCI (closely related to minor NCDs) and dementia who demonstrate executive dysfunction often show a markedly reduced IADL performance. Those with MCI frequently complete IADL tasks more slowly and less accurately than cognitively healthy individuals, displaying intermediate performance levels between healthy controls and individuals with mild AD [40]. Functional deficits in the IADL have also been observed in the early stages of cognitive decline, prior to the onset of dementia [44]. This led to the revisions in the diagnostic criteria for MCI, as proposed by Winblad et al. [17], to acknowledge that mild impairments in the IADL can manifest before a diagnosis of dementia is established.

### 4.3. IADL and Balance in NCDs

In the present study, balance impairments were found to significantly impact the IADL performance in both the minor and major NCD groups, as indicated by lower IADL scores among the participants with a reduced balance ability, affecting the complex tasks essential for independent living [45]. Previous studies have consistently linked balance and mobility impairments to decreased functional independence, emphasizing the importance of physical function in maintaining daily living activities [46]. Cognitive decline may contribute to the dysfunction in the bodily systems responsible for maintaining balance, supporting the notion that balance could serve as an early indicator of cognitive deterioration in older adults [47]. Additionally, the decline in cognitive abilities has been shown to affect gait control and postural adjustments, further exacerbating IADL impairments [45,48]. Furthermore, several studies have reported a strong association between executive functions and balance in older adults, regardless of the balance assessment method employed [49,50,51,52], with the relationship being particularly evident in dynamic balance and mobility-related tasks [52]. In individuals with minor NCDs, physical functions, including balance, have been found to significantly influence their IADL performance [47,53]. Among individuals with a diagnosis of MCI, IADL functioning has been associated with both cognitive functions (e.g., memory and executive functions) and physical capabilities, such as balance and mobility [20]. These findings suggest that improvements in balance may be associated with enhanced IADL performance. Moreover, Tinetti-balance scores have been shown to predict not only the mobility-related outcomes but also cognitive performance, reinforcing the link between motor function and higher-order cognitive abilities [54]. Individuals with Parkinson’s disease-related MCI (PD-MCI) demonstrate significantly more physical limitations in the IADL, poorer Tinetti-balance scores, and greater gait impairments compared to those with AD-related MCI (AD-MCI) [55]. Collectively, these findings highlight the critical role of balance impairments in restricting IADL performance. This relationship may differ depending on the underlying pathology and may be more pronounced in individuals with more severe forms of NCDs [56]. Given that IADL deficits frequently emerge early in cognitive decline and are a strong predictor of disease progression [34], and that physical activity has been shown to mitigate IADL impairments [46,57,58], interventions aimed at enhancing mobility and postural stability may be effective in slowing the functional decline [59,60].

### 4.4. Unique Predictors of the IADL in MinorNCD: TUG, Female Sex and Depression

In addition to the TINETTI-balance scores, the TUG test was identified as a significant predictor of IADL performance exclusively in the minor NCD group, with shorter TUG completion times associated with greater independence in the IADL. The TUG is a validated and widely used clinical measure of basic functional mobility, integrating the components of balance, gait, and lower limb strength [29]. A systematic review has confirmed its utility as a screening tool for the fall risk and functional decline in older adults [61]. The present findings align with the previous research indicating that shorter TUG times reflect preserved neuromuscular coordination and mobility, both of which are essential for the execution of complex daily tasks. Moreover, a reduced performance on the TUG has been independently linked to increased odds of IADL dependency in older populations [9,20].

Interestingly, the female sex was also associated with higher IADL scores, indicating greater independence in IADL tasks. Although women often report more functional limitations, this trend is frequently attributed to the higher prevalence of non-fatal disabling conditions such as arthritis and osteoporosis [62]. However, studies suggest that when the cognitive function is preserved, women may maintain or even surpass men in IADL tasks, potentially due to their lifelong social roles and adaptive coping strategies [20,21]. Lahav and Katz [63] emphasize the importance of examining gender-specific patterns in the IADL, while Khalagi et al. [9] confirmed that the female sex remains a significant factor in IADL outcomes even after adjusting for health and demographic variables.

In addition, lower levels of depressive symptoms were associated with better IADL performance. Depression has consistently been linked to reduced motivation, cognitive slowing, and decreased engagement in daily activities, all of which negatively affect functional autonomy in the IADL [64]. Subjective assessments of the IADL are particularly susceptible to mood-related bias, with depressive symptoms often leading individuals to overreport their functional limitations [20]. Of note, a depressive mood has been shown to independently predict IADL disability even when controlling for cognitive performance, as highlighted in multivariate analyses [21]. 

### 4.5. Strengths and Limitations

This study presents several notable strengths. First, it employed a comprehensive set of standardized assessment tools to evaluate the key domains of functioning: cognitive, motor, affective, and behavioral in individuals with NCDs. The inclusion of multiple validated scales enables a multidimensional analysis of the complex interplay between cognition, emotional, and neuropsychiatric profile, physical function, and caregivers’ burden. Additionally, the study was conducted in a real-world clinical setting, thereby enhancing the ecological validity of the findings.

Despite these strengths, the study had several limitations. Most notably, its cross-sectional design precluded the determination of the causal relationships between the cognitive, functional, and mobility-related factors. While the observed associations between IADL performance and cognitive, motor, and caregiver-related factors are robust, the directionality of these relationships remains unclear. It is plausible that functional decline may impact the cognitive and emotional status, or conversely, that cognitive impairments may lead to a diminished functional capacity. Longitudinal studies are warranted to clarify whether these factors are causal, reciprocal, or bidirectional and to track changes over time. Another limitation pertains to the sample size. Although sufficient for statistical analyses, the sample may not be large enough to generalize the findings to broader populations. Additionally, the potential selection bias must be acknowledged, as all the participants were recruited from a single university hospital-based dementia outpatient clinic. This recruitment strategy may limit the generalizability of the findings to other settings. The present study was conducted in Eastern Macedonia and Thrace, a region of Greece where family-based caregiving remains the predominant model of support for older adults with NCDs. In contrast to many Western European countries, institutional care (e.g., nursing homes or assisted living facilities) is relatively uncommon in Greece, particularly in non-urban regions. Cultural values emphasizing familial responsibility and intergenerational support often result in family members assuming primary caregiving roles, frequently without formal training or adequate psychosocial support. This sociocultural context may partially account for the elevated levels of caregiver burden observed in our sample and reflects a sociocultural environment in which independence in the IADL is highly valued. These cultural dynamics should be taken into consideration when interpreting the generalizability of the findings to other populations. Future multicenter studies with more diverse recruitment sources are necessary to improve the external validity. Finally, although objective measures were used, some instruments, such as the Lawton IADL Scale and the ZBI, rely on self-reports or caregiver input, which may introduce subjective bias.

### 4.6. Future Directions and Clinical Implications

Future research should utilize longitudinal study designs to monitor the changes in functional abilities over time and to establish the causal relationships among cognitive decline, mobility impairments, and IADL performance. Additionally, the inclusion of larger and more diverse samples from multiple clinical settings is necessary to enhance the generalizability of the findings. Integrating biomarkers, neuroimaging data, and advanced gait analysis techniques may further contribute to a more comprehensive understanding of the mechanisms underlying the functional decline in individuals with minor and major NCDs.

From a clinical perspective, these findings underscore the importance of a multidisciplinary approach for the assessment and management of individuals with NCDs. The strong associations between IADL performance and patients’ general cognitive status, executive functions in everyday problem-solving situations, and balance suggest that early interventions targeting both the cognitive and motor domains may help preserve independence and delay functional decline. Routine screening using standardized assessment tools can aid clinicians in identifying individuals at risk of functional deterioration. Furthermore, the study highlights the significance of caregiver burden, emphasizing the need for targeted support programs, psychoeducation, and respite care services to alleviate stress and promote caregiver well-being. The incorporation of personalized rehabilitation strategies, such as cognitive training, physical therapy, and balance exercises, may improve the functional outcomes and lower the risk of falls. Additionally, assistive technologies, home modifications, and structured daily routines can enhance independent living among individuals with NCDs, thereby contributing to health promotion in the aging population. Looking ahead, a patient-centered, integrative model that combines cognitive, physical, and psychosocial interventions will be essential for optimizing care and improving the quality of life for both patients and their caregivers.

## 5. Conclusions

This study highlights the associations between the cognitive status, executive dysfunction in everyday contexts, and balance impairments with IADL performance among individuals across the spectrum of NCDs, including both minor and major NCDs. Additionally, the findings underscore the additive role of the female sex and depressive symptoms in IADL functioning, particularly in individuals with minor NCDs. These factors may serve as the early indicators of functional vulnerability. However, given the cross-sectional design of the study, causal inferences cannot be made. Future longitudinal and interventional research is necessary to determine whether these variables can reliably predict functional decline or serve as effective targets for prevention and therapeutic strategies. While a multidisciplinary approach that integrates cognitive, physical, and psychosocial components shows potential, its efficacy must be confirmed through prospective studies. A more integrative, patient-centered model should be further explored to more effectively address the complex and evolving needs of individuals with NCDs.

## Figures and Tables

**Table 1:** Group characteristics for the total group of 117 patients as well as the two groups of MinorNCD and MajorNCD.

	Total Group	MinorNCD	MajorNCD	*p*-Value	Effect Size |d|
Age (years)	74.87 ± 8.42	72.07 ± 9.08	76.38 ± 7.69	0.008	0.53
Sex (M/F)	50/67	17/24	33/43	0.838	-
Education (years)	10.34 ± 4.72	11.76 ± 4.86	9.58 ± 4.50	0.017	0.47
Disease duration (years)	3.52 ± 2.44	3.15 ± 2.81	3.72 ± 2.21	0.224	0.24
ACE-III total score	56.59 ± 25.51	78.20 ± 15.25	44.93 ± 22.13	<0.001	1.66
FUCAS	67.04 ± 29.80	46.44 ± 13.18	78.16 ± 30.39	<0.001	1.23
GDS	3.32 ± 2.61	3.54 ± 2.61	3.20 ± 2.61	0.504	0.13
NPI total score	11.58 ± 13.76	7.02 ± 9.54	14.04 ± 15.06	0.008	0.52
TINETTI-gait	10.47 ± 2.82	11.07 ± 2.35	10.15 ± 3.01	0.070	0.33
TINETTI-balance	13.99 ± 3.38	14.81 ± 3.12	13.55 ± 3.45	0.054	0.38
TUG (sec)	9.25 ± 3.39	7.83 ± 3.02	10.02 ± 3.36	<0.001	0.68
IADL total score	4.21 ± 3.15	6.66 ± 2.19	2.90 ± 2.78	<0.001	1.45
Caregivers’ ZBI	22.34 ± 18.10	13.66 ± 14.12	27.03 ± 18.35	<0.001	0.79

*Notes*. M/F = male/female; ACE-III = Addenbrooke’s Cognitive Examination 3rd edition; FUCAS = Functional Cognitive Assessment Scale; GDS = Geriatric Depression Scale; NPI = Neuropsychiatric Inventory; TUG = Timed Up and Go Test; IADL = Instrumental Activities of Daily Living; ZBI = Zarit Burden Inventory. Differences in continuous variables between MinorNCD and MajorNCD were examined using *t*-test for independent samples whereas differences in sex distribution were tested using Chi square. Cohen’s |d| represents the magnitude of differences in continuous variables between MinorNCD and MajorNCD based on the following assumptions: d = 0.20, small effect size; d = 0.50, medium effect size; d ≥ 0.80, large effect size.

**Table 2:** Summary of regression analysis with IADL as dependent variable for the total group and separately for the MinorNCD and the MajorNCD groups.

	Unstandardized B (95% CI)	Standard Error B	Standardized Beta	*p*-Value
*Total group*	Model: F_3,116_ = 105.103; *p* < 0.001; adjusted R^2^ = 0.729			
ACE-III total score	0.041 (0.022, 0.061)	0.010	0.336	<0.001
FUCAS	−0.050 (−0.068, −0.033)	0.009	−0.477	<0.001
TINETTI-balance	0.208 (0.113, 0.304)	0.048	0.224	<0.001
				
*MinorNCD*	Model: F_3,40_ = 22.119; *p* < 0.001; adjusted R^2^ = 0.725			
Sex	1.119 (0.334, 1.904)	0.387	0.255	0.007
FUCAS	−0.087 (−0.118, −0.057)	0.015	−0.526	<0.001
GDS	−0.207 (−0.352, −0.062)	0.072	−0.247	0.007
TINETTI-balance	0.329 (0.210, 0.447)	0.058	0.470	<0.001
TUG	−0.185 (−0.318, −0.053)	0.065	−0.256	0.007
	
*MajorNCD*	Model: F_4,75_ = 44.335; *p* < 0.001; adjusted R^2^ = 0.634			
ACE-III total score	0.028 (0.003, 0.053)	0.013	0.226	0.027
FUCAS	−0.052 (−0.071, −0.033)	0.010	−0.571	<0.001
TINETTI-balance	0.124 (0.003, 0.244)	0.061	0.153	0.045

*Notes*. MinorNCD = minor neurocognitive disorder; MajorNCD = major neurocognitive disorder; FUCAS = Functional Cognitive Assessment Scale; ACE-III = Addenbrooke’s Cognitive Examination 3rd edition; GDS = Geriatric Depression Scale; TUG = Timed Up and Go Test. Only significant predictors for the final model are presented.

## Data Availability

The data presented in the study are available on request from the corresponding author due to privacy restrictions.
